# Home ventilation for patients with end-stage chronic obstructive pulmonary disease

**DOI:** 10.1097/SPC.0000000000000671

**Published:** 2023-08-23

**Authors:** Tim Raveling, Heidi A. Rantala, Marieke L. Duiverman

**Affiliations:** a Department of Pulmonary Diseases and Home Mechanical Ventilation; b Groningen Research Institute of Asthma and COPD (GRIAC), University of Groningen, University Medical Center Groningen, Groningen, The Netherlands; c Department of Respiratory Medicine; d Faculty of Medicine and Health Technology, Tampere University, Tampere, Finland

**Keywords:** advance care planning, COPD, non-invasive ventilation, quality of life

## Abstract

**Purpose of the review:**

The number of patients with end-stage chronic obstructive pulmonary disease (COPD) treated with chronic non-invasive ventilation (NIV) has greatly increased. In this review, the authors summarize the evidence for nocturnal NIV and NIV during exercise. The authors discuss the multidisciplinary and advanced care of patients with end-stage COPD treated with NIV.

**Recent findings:**

Nocturnal NIV improves gas exchange, health-related quality of life and survival in stable hypercapnic COPD patients. Improvements in care delivery have been achieved by relocating care from the hospital to home based; home initiation of chronic NIV is feasible, non-inferior regarding efficacy and cost-effective compared to in-hospital initiation. However, the effect of NIV on symptoms is variable, and applying optimal NIV for end-stage COPD is complex. While exercise-induced dyspnoea is a prominent complaint in end-stage COPD, nocturnal NIV will not change this. However, NIV applied solely during exercise might improve exercise tolerance and dyspnoea. While chronic NIV is often a long-standing treatment, patient expectations should be discussed early and be managed continuously during the treatment. Further, integration of advance care planning requires a multidisciplinary approach.

**Summary:**

Although chronic NIV is an effective treatment in end-stage COPD with persistent hypercapnia, there are still important questions that need to be answered to improve care of these severely ill patients.

Key Points

**Table:** 

Knowledge gaps	Clinical implications
What are the characteristics of patients with COPD that benefit the most from NIV?	Better selection of patients who benefit the most from chronic NIV will lead to better outcomes.
In patients with COPD treated with chronic NIV, does NIV-assisted exercise training have carryover effects to NIV-unassisted activities?	Results in better training programmes specifically for patients with persistent respiratory failure.
What are the expectations of patients with COPD prior to the initiation of NIV?	Improved education of patients and better management of expectations.
When is the right time to start ACP and how do patients on NIV experience ACP?	Better timing of ACP promotes palliative care decisions and avoids undesirable hospitalizations or treatments.

## INTRODUCTION

Patients with end-stage chronic obstructive pulmonary disease (COPD) experience severe and often disabling respiratory symptoms and poor exercise capacity, health-related quality of life (HRQL) and survival. At this stage, patients may develop chronic hypercapnic respiratory failure (CHRF). Chronic non-invasive ventilation (NIV) applied nocturnally at the patients’ home is an effective treatment for patients with CHRF. In recent years, NIV has gained acceptance as a treatment for patients with CHRF due to end-stage COPD. Guidelines now recommend NIV for patients with COPD and persistent hypercapnia ^1,2^. This has resulted in significant increases in the number of patients with COPD treated with home NIV. Nevertheless, chronic NIV poses several challenges to make it worthwhile. First, not all patients benefit from this treatment and there is a lack of knowledge about what predicts a beneficial effect. Second, titration of chronic NIV in this patient group is complex as ventilation needs to be improved in a diseased lung. Third, when starting chronic NIV in these end-stage patients, it often means that NIV is continued until death, therefore demanding a multidisciplinary approach integrated with advance care planning (ACP).

For this review, we have conducted a literature search on (a combination of) the following terms: ‘COPD’, ‘non-invasive ventilation’, ‘patient perspectives’, ‘patient experiences’, ‘palliative care’ and ‘ACP’. Relevant papers that were published in the past 3 years were selected, as well as older papers based on expert opinion. We first summarize the evidence for chronic NIV in patients with COPD. Second, we discuss intermittent NIV to relieve dyspnoea during exertion. In the second half of the review, we focus on the care for patients with COPD on chronic NIV. Is it useful to combine treatment options? What are the experiences of the patients with chronic NIV, and how can ACP be incorporated into the repeated contacts caregivers have with the patients? Finally, we discuss the need for integrating NIV with palliative care, including ACP.

## SUMMARY OF THE EVIDENCE FOR CHRONIC NON-INVASIVE VENTILATION

For a long period, chronic nocturnal use of NIV was not regarded to be beneficial in patients with CHRF due to COPD ^3–7^. However, in the last 15 years, several studies have shown meaningful benefits when chronic NIV targets normocapnia during the night, so-called high-intensity NIV ^8^. A recent Cochrane systematic review showed that in patients with COPD and persistent hypercapnia in a clinically stable disease phase, chronic NIV improves gas exchange ^9^. The improvement in gas exchange seems to be dependent on ventilatory settings and treatment adherence, with greater improvements in gas exchange achieved with higher inspiratory positive airway pressure and better treatment adherence ^9^. More importantly, patient-related outcomes, such as survival and HRQL, are improved by chronic NIV ^9–12^. Beneficial effects can be expected on complaints related to nocturnal hypoventilation, such as bad sleep, fatigue and mental performance ^13^. Unfortunately, NIV seems to be unable to improve dyspnoea during daytime while breathing without NIV ^8^. In contrast to stable hypercapnic COPD, the benefits of continuing NIV at home after an acute exacerbation are less evident ^8^. In this population, chronic NIV seems to prolong the admission-free survival, especially in patients with severe persistent hypercapnia more than 2 weeks after the acute event ^8,14^, but the effect on exacerbations and HRQL was lacking. This differential response between stable and post-exacerbation COPD has not been clarified yet. A possible explanation might be that in the post-exacerbation population, patients are extremely vulnerable and require multiple combined interventions to reduce exacerbations and improve their HRQL.

To date, patient-related factors associated with a beneficial effect have not been found yet. Recently, the interest in clinical phenotyping of patients with end-stage COPD has increased. Janssens *et al*.^15▪^ conducted a cluster analysis on a cohort of patients with COPD treated with chronic NIV and identified two distinct phenotypes. A respiratory phenotype, which included patients with a low body mass index and severe airway obstruction, and a systemic phenotype, which included patients with a higher body mass index and more comorbidities. Interestingly, survival was better in the systemic phenotype, but it is not clear whether this is due to a worse response to NIV or due to characteristics of the disease that are associated with a worse outcome. Future studies should focus on the identification of a phenotype associated with a beneficial effect on patient-centred outcomes (‘responder phenotype’) to further optimize the patient selection for NIV.

## NON-INVASIVE VENTILATION DURING EXERCISE FOR SYMPTOM RELIEF

Exercise training is a key component to maintain exercise capacity. A greater physiological response is achieved with training at high intensity, but patients with severe COPD are often incapable of achieving these high intensities due to dynamic hyperinflation and hypoxaemia, resulting in early lactatemia ^16^. Potential mechanisms by which NIV might improve exercise tolerance are respiratory muscle unloading, improving oxygenation due to better ventilation-perfusion ratio and reducing hyperinflation ^16^,^17^. A recent meta-analysis included 15 randomized controlled trials (RCTs), which studied the effect of NIV during a training exercise programme of 4–12 weeks ^18▪^, and concluded that after the training programme, the 6-min walking distance was higher in the group that trained with NIV and experienced lower fatigue during NIV use. However, there was no effect on dyspnoea. In most studies, NIV was well tolerated during exercise. The vast majority of the studies investigated patients naïve to NIV, limiting the generalizability to patients who are already familiar with nocturnal NIV. Two studies have investigated the acute effects of high-pressure NIV during exercise in patients who had already been initiated on nocturnal NIV. In both studies, NIV during exercise resulted in a better exercise capacity and reduction of dyspnoea ^19,20^, and the NIV group had less exercise-induced hypercapnia compared to the control group that trained with oxygen ^20^. So, to conclude, there is evidence that the use of NIV during exercise relieves dyspnoea and improves gas exchange and exercise capacity when applied with sufficient inspiratory pressures, but data are sparse. It remains unclear whether NIV has carryover effects on symptoms and endurance during exercise without NIV. Larger studies are needed in this specific population that incorporate NIV in exercise training programmes.

## MULTIDISCIPLINARY APPROACH

COPD is a heterogeneous disease, and hypercapnia is only one of the various treatable traits. Therefore, optimal treatment of patients with severe COPD should include a combination of treatment options, combined or initiated sequentially ^21^. Besides general recommendations like smoking cessation, nutritional support, sufficient physical activity and optimal pharmacological treatment, in this end-stage COPD population, bronchoscopic lung volume reduction, multidisciplinary pulmonary rehabilitation and/or lung transplantation might be worth considering ^22^. NIV may be of use for palliation of severe dyspnoea at end-stage disease without CHRF, but there is no literature to support this hypothesis. In some cases, chronic NIV may be useful as an add-on treatment. Severe hypercapnia is a relative contraindication for endobronchial valves, but valves may be considered after the initiation of NIV has improved gas exchange ^23,24^. Secondly, there is evidence that the initiation of NIV prior to a pulmonary rehabilitation is beneficial to the outcomes of the rehabilitation. The benefits achieved by rehabilitation on exercise capacity and HRQL seem to be better maintained when chronic NIV is subsequently continued at home, at least for patients with CHRF ^11,25^.

## INITIATION AND MONITORING

Historically there has been a high heterogeneity throughout Europe in the place where chronic NIV is initiated and where it is subsequently monitored ^26–29^. For many years, it was believed that the initiation of chronic NIV required hospital admission ^28^. This is especially true for patients with COPD, who generally are older and require higher ventilatory pressures to improve their ventilation. However, in-patient initiation and follow-up monitoring require substantial in-hospital healthcare resources, excessive costs, and often place a high burden on these severely ill patients.

In recent years, the interest in outpatient and home initiation and monitoring has increased and monitoring opportunities using telemonitoring are rapidly evolving. In a European survey conducted in 787 patients using NIV due to different diseases, the majority of patients would consider telemonitoring ^30^. Recently, three RCTs have shown that home initiation of NIV using extensive telemonitoring is feasible, safe and non-inferior to in-hospital initiation, both in patients with neuromuscular diseases and in patients with COPD ^29,31,32^. As may be expected, the cost of home initiation was over 50% lower compared to in-hospital initiation.

**Figure 1:**
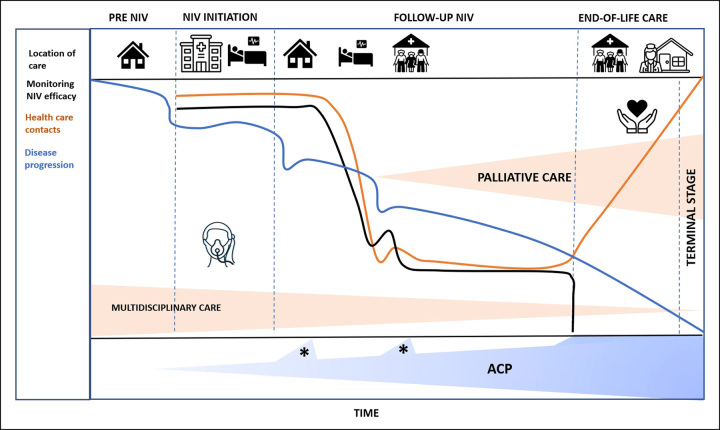
Comprehensive treatment of patients with chronic respiratory failure due to COPD in the end stage of disease. ACP, advanced care planning; NIV, non-invasive ventilation. Notes: *, represents an acute exacerbation of COPD. Location of care: home (
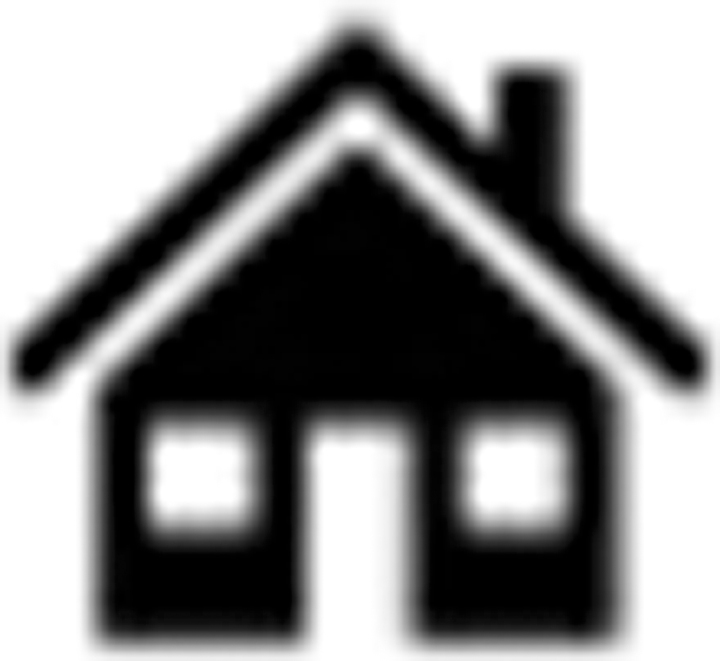
), in hospital (
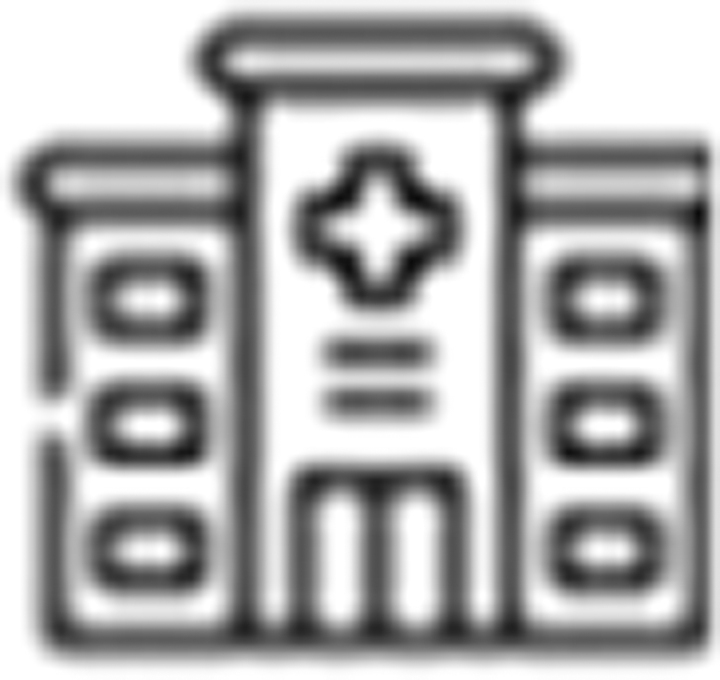
), outpatient based (
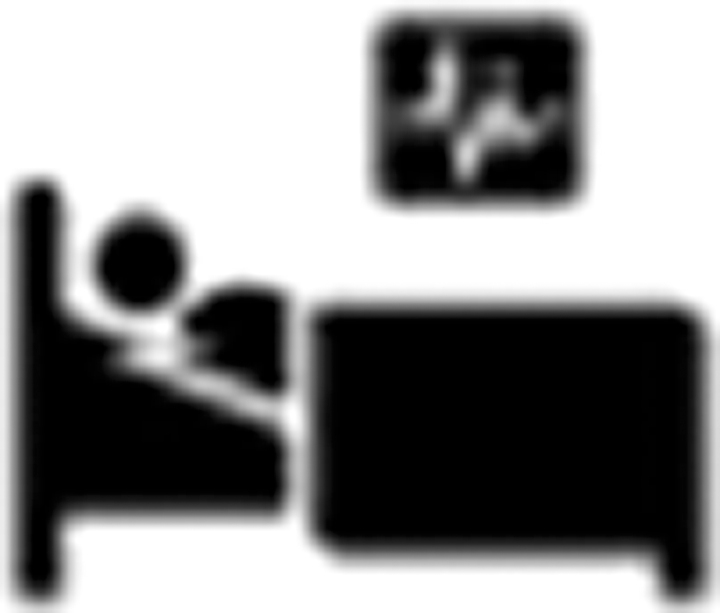
), nursing home or hospice (
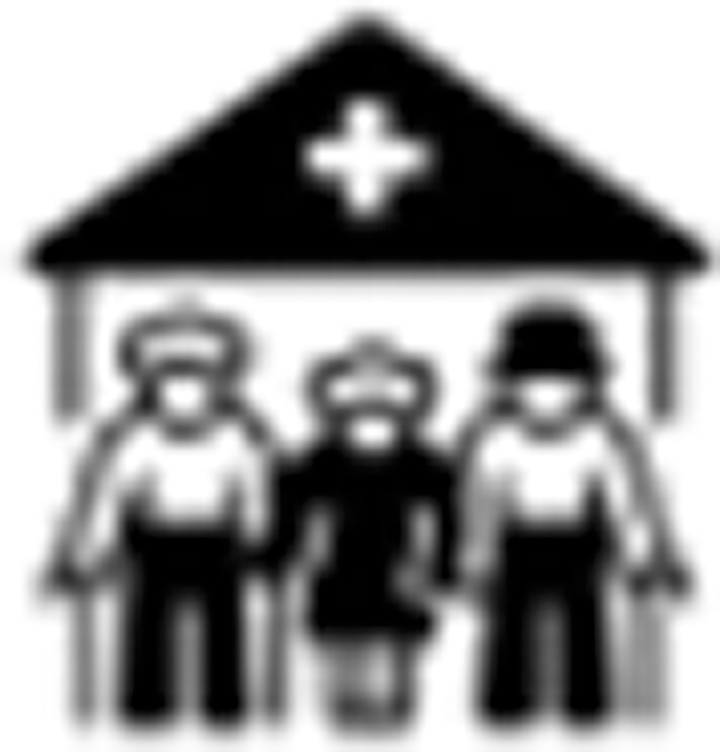
).

To ensure that NIV is applied effectively, and goals are achieved, regular monitoring of ventilator data (compliance, leakage, obstructions and patient–ventilator synchrony), nocturnal gas exchange and side effects is necessary ^33^. The frequency of monitoring is a subject of debate. Recently, the attention on more frequent (daily or weekly) remote monitoring of patients on home ventilation has greatly increased ^34▪^. Remote monitoring will personalize the follow-up, thereby preventing unnecessary and challenging hospital visits when patients are stable and intensifying the follow-up of deteriorating patients that might need intercurrent adjustments of ventilator settings (Fig. 1) ^34^,^35^. Moreover, healthcare systems taking care of those patients should be organized in a way that easy access to technical and clinical support is granted 24 h a day, 7 days a week, once needed.

For patients, the utmost goal of chronic NIV is to improve their HRQL. Both for clinical care and research, symptoms can be assessed systematically by using validated questionnaires, such as the Severe Respiratory Insufficiency questionnaire, the Maugeri Respiratory Failure questionnaire or the S3-NIV questionnaire ^36–39^. The S3-NIV questionnaire addresses both symptoms of respiratory failure, as well as side effects of the NIV. For use in clinical practice, both technological advances (like the development of an application) and the use of a short and self-administered questionnaire like the S3-NIV seem to be useful tools.

## PATIENT EXPERIENCES

For a successful and satisfying therapy, it is extremely important to manage patient expectations. In patients with progressive disabling diseases like severe COPD, it is of utmost importance to define goals of therapy. Most patients will strive for a reduction in symptoms, a better HRQL and a reduction in exacerbations or hospitalizations, and do not univocally strive for a longer life. Variable survival rates have been reported in patients with COPD on chronic NIV, but on average, survival is shorter compared to patients with slowly progressive neuromuscular disease or obesity-hypoventilation syndrome (median survival rates reported ranging from 2.7 to 4.4 years; 1-year survival reported ranges from 77 to 88%) ^10,40^. These findings stress the importance of discussing goals and expectations at the beginning of NIV and repeatedly during the follow-up.

There is limited literature on the experiences of patients once they have started chronic NIV, and there is no information on the expectations of patients prior to NIV initiation. The survey by Masefield *et al*.^30^ found that patients consider mask-related factors, such as leaks and comfort, as the important aspect of the treatment. A qualitative study by Caneiras *et al*.^41▪^ on the experiences of patients treated with home NIV (18 patients, 50% with COPD) found that most patients experience benefits of NIV on their symptoms. However, patients described the initial period as frightful and difficult due to adverse events and impact on their daily lives. Although, in general, these feelings resolved when benefits were subjectively perceived, the limitations on daily life persisted. This finding emphasizes the importance of a thorough patient education prior to initiation and of extensive support during the initial initiation. Unfortunately, predicting the individual’s response to NIV remains difficult, as a ‘responder phenotype’ has not been defined. This complicates the management of the expectations of the patients.

## ADVANCE CARE PLANNING IN PATIENTS WITH CHRONIC OBSTRUCTIVE PULMONARY DISEASE TREATED WITH CHRONIC NON-INVASIVE VENTILATION

A majority of patients with COPD who have successfully started on NIV will be ventilated until they die. This means that a repeated discussion is needed with your patients on how to deal with end-stage disease. At end stage, symptoms often deteriorate resulting in increasing hours of ventilator use, which require more frequent healthcare contacts. We suggest a shift of focus from monitoring of efficacy to comfort of NIV (Fig. 1). Also, it is extremely important to discuss expectations, current experienced HRQL and integrate ACP. ACP has been defined as an individuals’ ability to define goals and preferences for their future treatment and discuss these with their close ones and healthcare professionals ^42^. Even though ACP is recommended by several guidelines ^22^,^43^,^44^, it is still uncommon in patients with severe respiratory diseases ^45,46^. Palliative care is part of ACP and was recently defined in patients with COPD as a holistic, multidisciplinary, person-centred approach aiming to control symptoms, improve HRQL and support patients’ informal caregivers ^44▪▪^. Although it was based on low to very low quality of evidence, there were no unsolicited effects due to palliative care interventions. Limiting factors for performing ACP seem to be lack of time and the difficulty in predicting the disease course ^47,48^. Further, even though palliative care specialists have good knowledge of ACP, they lack information on ACP specifically for patients with end-stage COPD ^49▪^. Still more research is needed on how to integrate ACP into routine care for patients with end-stage COPD ^44▪▪^.

With a progressing disease, ACP should be integrated early enough to know patients’ perspectives. Even though starting chronic NIV might lead to improved HRQL, more stable disease and sometimes better survival, palliative care should be part of the treatment as patients at this stage suffer from wide variety of severe symptoms ^50,51^. Unfortunately, in a study performed in Finland, end-of-life decisions were made in only 39% of the patients with end-stage COPD and only 23% of the patients in their cohort died at home ^52^. The likelihood to die at home may be increased by palliative care ^53^. Besides, specialist palliative care consultations were associated with reduced emergency room visits and hospital days during the last year of life in patients with end-stage COPD ^54^. This highlights the importance of discussing not only intubation and resuscitation orders but also to take a broader view on advance care discussions to avoid unnecessary hospitalization. Given the poor survival of patients with COPD treated with chronic NIV, ACP should be started at least when NIV is initiated and should be discussed continuously throughout the treatment (Fig. 1) ^55,56^. This ensures a timely progress to palliative care for comprehensive symptom palliation, also concerning symptoms beyond NIV.

The difficult question is when to proceed to the terminal stage. Increasing hours of NIV (e.g. during the daytime), worsening of gas exchange and symptoms despite high ventilatory settings and frequent hospitalizations are signs that may indicate the start of the terminal stage. At this stage, patients may choose to stop NIV as it is not leading to sufficient improvement anymore whilst others stick to nocturnal use. Some patients will increase their ventilator use up to 24 h per day to relieve symptoms and still experience a satisfactory HRQL. It remains key to provide care from a multidimensional perspective; by combining non-pharmacological (including NIV) and pharmacological treatment options to reduce symptoms, a satisfactory end-of-life period should be achievable.

## CONCLUSION

In patients with CHRF due to COPD, chronic NIV is an effective treatment to improve gas exchange, HRQL and survival when patients are clinically stable. Unfortunately, characteristics of patients that will benefit the most have not been identified, which complicates the selection of the most suitable patient and the management of patient expectations. The initiation of NIV should be a trigger to initiate ACP, which can still be implemented broader to promote a better symptom palliation, end-of-life care and avoid undesirable hospitalizations or treatments. As patient numbers are expected to rise in the coming years, answering these questions will certainly result in better care for these severely ill patients.
